# The prevention and treatment of cognitive decline and dementia: An overview of recent research on experimental treatments

**DOI:** 10.4103/0019-5545.44900

**Published:** 2009

**Authors:** Chittaranjan Andrade, Rajiv Radhakrishnan

**Affiliations:** Department of Psychopharmacology, National Institute of Mental Health and Neurosciences, Bangalore - 560 029, India

**Keywords:** Mild cognitive impairment, Alzheimer's disease, dementia, cognition, elderly

## Abstract

The prevention and treatment of cognitive impairment in the elderly has assumed increasing importance in an aging population. This article presents a qualitative review of recent research on experimental interventions for the prevention and treatment of mild cognitive impairment and Alzheimer's disease in elderly subjects. Interventions addressed range from lifestyle measures to pharmacological treatments. Epidemiological studies suggest that dietary measures, physical exercise, and mental activity may reduce the risk of cognitive impairment and Alzheimer's disease in elderly subjects. Statins may protect against incident dementia, and lithium may convey similar benefits to bipolar patients. Ginkgo appears ineffective as a primary preventive measure. Donepezil but not Vitamin E may benefit persons with mild cognitive impairment. Experimental treatments potentially useful for Alzheimer's disease include dimebon, PBT2 and etanercept; the safety and efficacy of the Alzheimer's vaccine remains to be proven, and growth hormone secretagogue and tarenflurbil are likely ineffective. Herbal treatments merit study in elderly subjects with cognitive syndromes.

## INTRODUCTION

Life expectancy is increasing as a result of advances in medical science and the availability of better healthcare services; the proportion of elderly persons in the general population is therefore rising. As the risk of dementia increases with increasing age, the number of persons with dementia in the general population is also rising. For example, Kokmen *et al.*[1] found that the cumulative incidence of dementia per 1000 in the USA rose from 3.5 in the 65-69 year age group to 72.8 in those aged 85 years and older; and the incidence per 1000 in another USA study were 2.2 in the 65-74 year age group and 26.0 in those aged 85 years and older.[2]

In a more recent longitudinal study, the prevalence of dementia in the USA was reported at 13.9% among individuals aged 71 years and older.[3] Alzheimer's disease (AD) accounted for 69.9%, vascular dementia (VaD) for 17.4%, and other types of dementia (such as “dementia, undetermined etiology,” Parkinson's dementia, normal-pressure hydrocephalus, frontal lobe dementia, alcoholic dementia, traumatic brain injury, and Lewy body dementia) accounted for 12.7% of all cases of dementia.

In a study by the 10/66 Dementia Research Group, global estimates for 2001 showed that, worldwide, about 24.3 million persons suffered from dementia; and 60.1% of all people with dementia lived in developing countries.[4] In India, the prevalence of dementia lies in the range of 1.8% to 3.6% among individuals aged 60 to 65 years and above (the cut-off age varied across studies)[5–7]

All degenerative disorders are presumed to begin insidiously and progress gradually. The detection of an early stage of partial symptomatology may then offer an opportunity for secondary prevention. In the context of dementia, Mild Cognitive Impairment (MCI) may represent this incipient stage. MCI refers to newly acquired cognitive deficits which are more severe than expected (based on age and educational background) and which do not as yet cause social or occupational impairment. MCI has been proposed as a transition between normal, age-associated cognitive change and early dementia.[8]

The concept of MCI has attracted controversy.[9,10] Although the MCI construct was initially limited to mild memory Impairment (amnestic MCI),[8] it is today accepted that it is probably more heterogeneous and includes amnestic, nonamnestic, and single vs. multiple domain subtypes. The rates of progression to dementia and the stability of the MCI diagnosis vary widely, depending on the study setting, recruitment methods and operational criteria.

The prevalence of MCI was 5.3% in Japan; the yearly rate of progression of MCI to dementia was about 4%.[11] The rate of amnestic MCI in the USA, in a cohort of 1,248 individuals with mean age of 74.6 (± 5.3) years, was about 3-4%.[12] In a Swedish cohort, among those aged 75-95 years, the rates of amnestic MCI, single domain non-memory MCI and multidomain MCI were 2.1%, 7.2% and 1.8% respectively, using standard definitions; these rates were almost doubled when the criterion for normal cognitive functioning was removed in the modified definitions.[13] The rate of MCI in India among those aged 50 years and older was 14.9% (95% CI, 12.2-18.0); amnestic MCI accounted for 6.0% and multidomain MCI for 8.9%.[14]

MCI may predict Alzheimer's disease (AD). The rate of progression to AD was highest for multidomain MCI (HR, 23.6; 95% CI, 9.3-60.1) followed by amnestic MCI, modified criteria (HR, 17.9; 95% CI, 6.8-46.9).[13]

### Scope of the present article

This article will present in separate sections a qualitative overview of current research on experimental treatments related to the following themes:

1. The prevention of mild cognitive impairment and Alzheimer's disease
2. The treatment of mild cognitive impairment
3. The prevention of the progression of Alzheimer's disease
4. The treatment of cognitive symptoms in elderly persons with and without cognitive syndromes.

For the most part, the presentation will feature selected original studies in order to provide the reader with a research-based perspective followed by a critical appraisal. As there is inevitably some overlap across sections, treatments are classified in the most appropriate section with an acknowledgement of the overlap.

## THE PREVENTION OF MILD COGNITIVE IMPAIRMENT AND ALZHEIMER'S DISEASE

Currently approved medications for AD have a discouragingly small effect on cognition and on disease progression. In a Cochrane Review of 10 randomized, double blind, placebo-controlled trials, the improvement with 6 months of cholinesterase therapy was, on average, just 2.7 points in the middle of the range of the 70-point ADAS-Cog Scale.[15] Pharmacotherapy will hence need to be directed at primary prevention strategies because neurodegeneration may be impossible to reverse. MCI is therefore a logical target for early intervention against AD, and age-related cognitive impairment (not amounting to MCI) may hold even greater promise. However, the implementation of preventive measures in cognitively healthy individuals offers the best hope against the onset of neurodegeneration.

There is a large body of literature on primary prevention strategies for AD. Modifiable risk factors for AD are well known; these include smoking, hypertension, high homocysteine levels, type 2 diabetes, insulin resistance, hypercholesterolemia, and obesity. Higher education, physical exercise, and mental exercise are well established as important pro-cognitive attributes and behaviors. Dietary measures, such as high intake of fish, fruit and vegetables suggest a positive role for omega-3 fatty acids (docosahexaenoic acid and eicosapentaenoic acid), antioxidants (vitamin E and flavonoids), and B group vitamins such as folate, B6, and B12. Certain recent studies in these areas will be briefly examined. When reading these studies, readers should keep in mind that epidemiological data suffer from inescapable confounds. For example, more intelligent persons are simultaneously more likely to pursue higher studies and less likely to develop dementia because of higher cognitive reserve; therefore, low education may merely be a marker of dementia risk and not an independent risk factor.

### Fish, fruit, and vegetable consumption may lower the risk of Alzheimer's disease

Epidemiologic studies suggest that a diet rich in fish is associated with less cognitive decline[16] and with a decreased risk of Alzheimer's disease.[17] The benefits may be specific to the intake of fatty fish, such as tuna, and may be evident only in those who are negative for the apolipoprotein E (ApoE) epsilon 4 allele.[18] Regular fruit and vegetable intake, even in the form of juices, has also been associated with a decreased risk of Alzheimer's disease.[19]

Another recent study on the subject was that of Barberger-Gateau *et al.*[20] These authors examined data from 8,085 nondemented participants aged 65 and above, recruited in the Three-City cohort study in Bordeaux, Dijon, and Montpellier (France) in 1999-2000. All subjects had at least one re-examination over 4 years; the follow-up rate was 89.1%. During follow up, there were 281 cases of incident dementia; of these, 183 were diagnosed with Alzheimer's disease. The data were analyzed with adjustments for sociodemographic and vascular risk factors.

Daily consumption of fruit and vegetables was found to be associated with a 28% decrease in the risk of all-cause dementia (HR, 0.72; 95% CI, 0.53-0.97). Weekly consumption of fish was associated with a 35% reduced risk of Alzheimer's disease (HR, 0.65; 95% CI, 0.43-0.99). Weekly consumption of fish was associated with a 40% reduction in the risk of all-cause dementia in subjects who were negative for the allele (HR, 0.60; 95% CI, 0.40-0.90). The regular use of omega-3 rich oils was associated with a decreased risk of all-cause dementia which, however, did not reach statistical significance. The regular consumption of omega-6 rich oils uncompensated by consumption of omega-3 rich oils or fish was associated with a doubled risk of dementia among subjects who were negative for the allele (HR, 2.12; 95% CI, 1.30-3.46). There were no significant associations between consumption of corn oil, peanut oil, lard, meat, or wine and the risk of dementia.

Thus, these findings suggest that, in men and women aged 65 years and above, the daily intake of fruit and vegetables and the weekly intake of fish are associated with a 30%-40% decrease in the risk of dementia and Alzheimer's disease across a 4-year follow-up period. At least some of the benefits are restricted to subjects who are negative for the allele.

The allele is a risk factor for Alzheimer's disease. This allele has a low prevalence in the general population. Therefore, the regular intake of fish, fruit, and vegetables is important for most people, and has important public health significance for the primary prevention of dementia. The results of studies such as this and others (e.g., Huang *et al.*, 2005[18]) imply that the negative impact of the allele offsets the positive impact of dietary measures in influencing the risk of dementia.

Animal studies show that diets rich in omega-3 fatty acids have many neurophysiological, neurochemical, cognitive, and other central nervous system (CNS) benefits. The benefits of dietary fish are likely mediated by omega-3 fatty acids, especially docosahexaenoic acid (DHA). Fish is a direct dietary source of DHA. DHA is also synthesized in the body from linolenic acid and eicosapentaenoic acid (EPA). DHA is an important component of membrane phospholipids in mitochondria, synaptosomes, and synaptic vesicles in the brain. High levels of DHA are found in the cerebral cortex.[17]

Canola oil, flaxseed oil, and walnut oil are rich in omega-3 fatty acids. In contrast, sunflower oil and grape seed oil are rich in omega-6 fatty acids. Of note, an inescapable source of confound in epidemiological studies such as this is that a healthier diet may be a consequence or correlate of better cognitive health in elderly individuals, and not the cause.

### Other recent studies on omega-3 fatty acids, fish intake, and cognition in the elderly

Several other recent studies have also examined the role of omega-3 fatty acids in the context of cognition in the elderly. These studies are briefly examined.

### Nurk et al. (2007)[21]

These authors described a cross-sectional study of the relationship between cognitive performance and seafood intake. The sample comprised 2031 subjects (55% women), aged 70-74 years, who were recruited from the general population in Western Norway. These subjects completed an extensive battery of cognitive tests; impairment was defined as cognitive scores in the worst tenth of the performance range.

Important findings were that subjects with a mean intake of >10 g/day of fish and fish products (*n* = 1951) had significantly better mean test scores and a lower prevalence of impaired cognitive performance than subjects whose intake was < 10 g/d (*n* = 80). The associations between cognition and total intake of seafood were strongly dose-dependent; the maximum effect was observed at an intake of approximately 75 g/d. Most individual cognitive functions showed significant associations with fish intake. The effect was stronger with nonprocessed lean fish and fatty fish.

### van Gelder et al. (2007)[22]

These authors examined the associations between fish consumption, the intake of EPA and DHA omega-3 fatty acids from fish and other foods and subsequent 5-year cognitive decline. The sample comprised 210 men, aged 70-89 years, in the Zutphen Elderly Study in the Netherlands.

Important findings were that persons whose diet included fish had significantly less cognitive decline than those whose diet did not include fish. Higher intake of EPA and DHA were associated with less cognitive decline in a linear, dose-dependent fashion.

Thus, the findings of Nurk *et al.*[21] and Gelder *et al.*[22] suggest that elderly subjects who eat more fish and fish products have better cognitive performances across a range of cognitive domains; the effect is dose-dependent. Elderly subjects who eat fish and who consume more omega-3 fatty acids in their diet suffer less cognitive decline across 5 years; the effect is again dose-dependent.

Using data from a Dutch study (FACIT) which compared folic acid with placebo in 807 subjects aged 50-70 years, Dullemeijer *et al.*[23] showed that higher plasma very long chain omega-3 polyunsaturated fatty acid proportions were associated with less decline in speed-related cognitive domains across 3 years. Using data from an American study on atherosclerosis risk (ARIC) in 2251 healthy, white subjects aged 50-65 years, Beydoun *et al.*[24] showed that, across a 9-11 year follow-up, higher levels of omega-3 fatty acids reduced the risk of decline in verbal fluency, especially in hypertensive and dyslipidemic subjects. There were, however, no significant findings related to psychomotor speed and delayed word recall.

### Tea intake may protect against cognitive decline in the elderly

The regular intake of tea has been associated with a number of health benefits, including a reduced risk of cardiovascular, cerebrovascular, cognitive, and other disorders. A recent study was that of Ng *et al.*[25] The sample comprised community-dwelling Chinese adults aged 55 years and older in the Singapore Longitudinal Ageing Studies cohort. Tea consumption was assessed at baseline. The mini-mental state examination (MMSE) was administered at baseline and again, 1-2 years later (median, after 16 months). Cognitive impairment was defined as an MMSE score of 23 or less; and cognitive decline was defined as a 1-point or greater drop in the MMSE score at follow up.

A cross-sectional analysis was performed on the baseline data from 2501 subjects. A longitudinal analysis was performed on follow-up data from 1438 cognitively intact subjects. Analyses controlled for important variables that were potential confounders.

*At baseline*, total tea intake was dose-dependently associated with a lower prevalence of cognitive impairment. Relative to rare or no tea intake, low tea intake reduced the risk of cognitive impairment by 44%; medium intake by 55%; and high intake by 63%. *At follow-up*, tea intake was dose-dependently associated with a decreased risk of cognitive decline. The benefits of tea were most apparent for black (fermented) and oolong (semi-fermented) teas, the predominant types consumed by the subjects in the study; the benefits were, again, dose-dependent. The intake of green tea was associated with a lower prevalence of cognitive impairment but not cognitive decline; however, this analysis was based on small numbers. There was no significant association between coffee intake and cognitive status at baseline or follow-up. Thus, these data suggest that the regular intake of tea (but not coffee) is dose-dependently associated with a lower risk of cognitive impairment and cognitive decline.

Causality cannot be determined from epidemiological data. For example, in this study higher tea consumption at baseline was associated with younger age, male predominance, higher education, higher levels of physical and social activity, greater intake of fruit and vegetables, higher alcohol consumption, and lower prevalence of depression; most of these variables are known to be associated with better cognitive status. However, causality can sometimes be inferred from indirect evidence. For example, after controlling for the above-mentioned confounding variables in a logistic regression analysis, the dose-dependent effects of tea and (more importantly) the absence of benefits with coffee (a control beverage) suggest that tea drinking may indeed have had a protective effect.

### Physical exercise may reduce the risk of dementia

A large body of epidemiological literature suggests that higher levels of intelligence, education, occupational attainment, and participation in intellectual and leisure activities independently reduce the risk of Alzheimer's disease.[26] Much evidence suggests that higher levels of physical activity also reduce the risk of dementia.

In a recent Italian study, Ravaglia *et al.*[27] examined a prospective, population-based cohort of 749 subjects, aged 65 years and older, who were cognitively normal at baseline. Baseline physical activity was measured in the context of walking, stair climbing, moderate activities, vigorous activities, and total physical activity. During an average of 3.9 years of follow up, 54 subjects developed Alzheimer's disease, and 27 developed vascular dementia. Comparisons were drawn between subjects in the upper and lower tertiles of physical activity; these analyses were adjusted for sociodemographic and genetic risk factors.

The risk of vascular dementia was about 70-75% lower for walking, moderate physical activity, and total physical activity. These findings remained significant even after adjusting for vascular risk factors and overall health status. However, after adjusting for confounding variables, no significant relationship was found between exercise variables and the risk of Alzheimer's disease.

In another recent study, Larson *et al.*[28] followed 1740 Americans, aged 65 years and older, for a mean of 6.2 years. The adjusted risk of dementia was nearly 40% less in those who exercised at least thrice a week. The advantage for exercise remained significant in analyses that examined the risk of Alzheimer's disease. Differences in lifestyle and other risk factors between American and Italian subjects may explain the differences in the findings between the studies of Larson *et al.*[28] and the Ravaglia *et al.*[27]

How might exercise protect against dementia? Exercise facilitates neuroplasticity in the brain. For example, Stranahan *et al.*[29] showed that running increased dendritic spine density in the entorhinal cortex and hippocampus of adult rats. This effect was observed not only in granule neurons of the dentate gyrus but also in CA1 pyramidal neurons, and in layer III pyramidal neurons of the entorhinal cortex. Potential consequences of these changes are improved synaptogenesis and enhanced hippocampal functioning.

Reviewing the literature, Lange-Asschenfeldt and Kojda[30] concluded that besides facilitating neuronal plasticity, exercise may act through an increase in the vascular reserve; as vascular risk factors have been implicated in Alzheimer's disease, neurodegeneration, presumably, is less likely if there is a healthy circulation and a large collateral circulation in the brain.

Van Uffelen *et al.*[31] found that adherence to a regular exercise regime was important for the mediation of cognitive benefits. On a more somber note, a recent Cochrane Review which included two randomized controlled trials in its meta-analysis concluded that there was insufficient evidence to determine the effectiveness of physical activity programs in managing or improving cognition, general functioning, behaviour, depression, and mortality in people with dementia.[32]

### Benefits of cognitive training in the elderly

Epidemiological studies suggest that persons who are mentally active are at a lower risk of cognitive decline and dementia in old age.[33,34] What is not clear, however, is whether there is a cause-effect relationship; does mental activity create a mental reserve and thereby protect the brain from cognitive decline, or are people who are biologically at lower risk of cognitive decline more mentally active because of a greater mental reserve? The issue can only be resolved through prospective, controlled studies, and the 2-year and 5-year outcomes of such a study were reported by Ball *et al.*[35] and Willis *et al.*,[36] respectively.

Briefly, the sample comprised 2832 physically and mentally healthy, independently-living elderly volunteers (age, 65-94 years; mean, 74 years) who were randomized into memory training (*n* = 711), reasoning training (*n* = 705), processing speed training (*n* = 712), or control (*n* =704) groups. Memory training sought to enhance verbal episodic memory skills. Inductive reasoning training sought to improve the ability to solve problems that follow a serial pattern. Processing speed training sought to improve visual search and identification skills. There was no intervention in the control group.

The training to the three intervention groups was provided in ten 60-75 minute group sessions distributed across 5-6 weeks. The training comprised instruction in cognitive strategies as well as actual exercises. Importantly, the topics for training were abstract as well as those related to everyday life.

Eleven months after the initial training, a random sample of 60% of the subjects in each intervention group was selected for four 75-minute booster sessions of training distributed across 2-3 weeks. Similarly, 35 months after the initial training, selected subjects received 4 more booster sessions of training. The booster sessions were conducted in the same domains in which the subjects had originally been trained.

Eighty percent of participants were retained at a 2-year follow-up, and 67% at a 5-year follow up. Assessments were conducted by raters who were blind to the intervention status of the subjects. Self-reported and performance-based measures of daily functioning and cognitive abilities were also obtained; these, of course, were non-blind.

At the end of the training phase, each intervention significantly improved the targeted cognitive ability: speed of processing improved in 86%, reasoning in 74%, but memory in only 26% of the persons in the respective groups. Booster training significantly enhanced training gains, but only in the reasoning and speed of processing groups. In each of the 3 groups, cognitive improvements with training remained significant at the 2-year follow-up. However, improvements in the tested abilities notwithstanding, there was no difference in instrumental activities of daily living in any of the four groups.

On formal testing, each intervention showed persistent gains in the targeted cognitive ability at the 5-year follow-up. The effect sizes were 0.23 for memory, 0.26 for reasoning, and 0.76 for speed of processing. Booster training improved tested performance in the targeted cognitive ability in the reasoning (effect size, 0.28) and speed of processing (effect size, 0.85) groups but not in the memory group. Relative to controls, only the reasoning group reported significantly less difficulty in instrumental activities of daily living (effect size, 0.29). In the speed of processing group, the booster training improved performance on a functional measure of everyday speed of processing (effect size, 0.30). Everyday performance did not improve with booster training in either of the other two groups. Booster training did not improve problem-solving or instrumental activities of daily living in any of the 3 training groups.

These results suggest that elderly subjects who are group-trained in memory, reasoning, or speed of processing skills show persistent gains in these specific domains at 2- and 5-year follow-up; training, however, results in gains only in the domain in which the training was conducted. Booster sessions of training months or years after the original training further enhance training-specific skills. The greatest benefits are observed for training related to speed of processing. The least benefits are observed for training related to memory. Effects sizes are small for training in reasoning and memory and large for training in speed of processing. With minor exceptions, the cognitive improvements do not generalize into improvements in everyday life.

In this study, the magnitude of neuropsychological gain at 2 years was encouraging; it was equivalent to a protection from 7 to 14 years of cognitive decline in elderly persons without dementia. The lack of translation of cognitive gains into functional gains at 2 years was disappointing. In explanation, a large proportion of the sample was already functioning at a high level; therefore, no further improvement could be expected. Whereas the reasoning group showed gains in everyday life at a 5-year follow-up, consideration must be paid to the limitation that this finding was based on self-reports that were nonblind. Memory training was associated with the least benefits. This implies either that memory is a domain that will inevitably show decline or that the memory training conducted by the authors was not intensive enough. Generalization to everyday life was poor in all three groups. This implies that subjects may need greater training in practical skills than in cognitive abilities or that subjects may need greater instruction in how to apply their training to everyday situations. The benefits were observed specific to the targeted cognitive domains; subjects trained in one domain did not improve in the other domains. A physical parallel is that the exercise of one muscle will strengthen only that muscle and not others. The most important take-home message is that a low-intensity cognitive training program can result in cognitive as well as some real-life benefits; these are sustained for up to 5 years afterwards and may be enhanced with booster training.

### Statin use may protect against incident dementia

Some epidemiological data suggest that the use of statins is associated with a decreased risk of incident dementia.[37,38] A recent study was that of Cramer *et al.*[39]

The data were drawn from a population-based cohort study of 1789 elderly Mexican Americans. All subjects had cognitive and clinical evaluations performed every 12 to 15 months. There were 1674 subjects who were free of cognitive impairment at baseline. Of these, 27% used statins at some time during the course of the study. Across 5 years of follow-up, 130 subjects developed dementia or cognitive impairment without dementia. After adjusting for education, smoking, presence of ApoE epsilon 4 alleles, and previous history of diabetes or stroke, the use of statins was found to be associated with a halved risk of dementia or cognitive impairment without dementia (HR, 0.52; 95% CI, 0.34-0.80). This means that, across a 5-year follow-up period, cognitively healthy elderly subjects who use statins enjoy a halved risk of dementia or cognitive impairment without dementia.

How may the regular use of statins reduce the risk of Alzheimer's disease? The enzymes beta- and gamma-secretase cleave amyloid precursor protein and form beta amyloid, which in turn forms the amyloid plaque that characterizes Alzheimer's disease. Only a small quantity of amyloid precursor protein follows this pathway; the rest is cleaved by alpha-secretase to form non-toxic products. As statins inhibit beta-secretase and activate alpha-secretase, they decrease the beta amyloid load.

In animal studies, statins have been shown to reduce beta amyloid levels.[40] Statins may also reduce the risk of dementia and cognitive impairment by modifying the vascular risk factors that have been implicated in both vascular dementia and Alzheimer's disease. Several other studies have also found statins to be associated with a decreased risk of Alzheimer's disease and dementia.[37,38] These encouraging results should be balanced against the negative results of other studies.[41,42]

### Lithium may protect against dementia in bipolar patients

Bipolar disorder is associated with increased risk of dementia.[43] Lithium has been shown to stimulate neuroplasticity in the brain.[44] Lithium also inhibits the formation of both beta amyloid and hyper phosphorylated tau protein.[45,46] So, might lithium reduce the risk of dementia in bipolar patients?

Nunes *et al.*[47] compared the prevalence of Alzheimer's disease between 66 lithium-treated elderly bipolar patients and 48 similar patients who had not received lithium recently. The prevalence of dementia in the whole sample was 19% relative to just 7% in an age-comparable population. Alzheimer's disease was diagnosed in just 3 (5%) of the patients who were receiving long-term lithium treatment; in contrast, Alzheimer's disease was diagnosed in 16 (33%) of the patients who were not on lithium (*P* < 0.001). This advantage for lithium was statistically significant. Although this study was very small, it suggested that lithium treatment may decrease the risk of Alzheimer's disease in bipolar patients to age-adjusted levels in the general population.

In a large, nationwide observational cohort study conducted in Denmark, Kessing *et al.*[48] compared lithium-treated patients with a random sample of 30% of the general population. There were 16,238 persons who had purchased lithium at least once, and 1,487,177 persons in the general population who had not purchased lithium. The lithium patients had an increased risk of dementia relative to the population controls (RR, 1.47; 95% CI, 1.22-1.76); this probably represents the increased risk of dementia in bipolar patients. Relative to patients who received only one prescription for lithium, those who received repeated prescriptions had a 15-34% lesser risk of dementia; this suggests that the continued use of lithium was associated with a lower risk. Interestingly, the relationship between number of lithium prescriptions and risk of dementia was not dose-dependent; and in different subanalyses, the benefits associated with lithium use were generally significant for non-Alzheimer dementias and total dementia, and not for Alzheimer's disease. Importantly, anticonvulsant use was associated with an increased risk of dementia, though, strictly speaking, the data are not comparable because anticonvulsants are primarily used to treat epilepsy.

Negative results have also been published. Dunn *et al.*[49] described a case-control study using data from the General Practice Research Database in the UK. They found that patients who received lithium had a higher risk of dementia relative to those who did not (OR, 1.8; 95% CI, 1.1-2.8); there was a trend towards increasing risk with increasing numbers of lithium prescriptions.

At present, therefore, the data suggest potential benefits, but recommendations cannot as yet be made.

### Ginkgo biloba is ineffective in primary prevention

Many studies have found cognitive benefits with a standardized extract of Ginkgo biloba after intermediate- to long-term administration to patients with Alzheimer's disease.[50] However, several recent, large studies have found Ginkgo no better than placebo.

Dodge *et al.*[51] described a 42-month, randomized, double-blind, placebo-controlled study, which examined whether Ginkgo could delay the progression to cognitive impairment in 118 cognitively intact persons aged 85 years and above. Ginkgo was ineffective on the primary outcome measures in the sample as a whole; however, Ginkgo reduced memory decline and progression to cognitive deterioration in those who showed acceptable adherence to medication. Disconcertingly, Ginkgo was associated with a significantly elevated risk of transient ischemic attacks and stroke.

McCarney *et al.*[52] conducted a 6-month, community-based, pragmatic, randomized, double-blind, placebo-controlled trial of Ginkgo biloba in 176 patients with early dementia. Ginkgo was no better than placebo on ADAS-Cog and patient- and caregiver-rated quality of life outcomes.

DeKosky *et al.*[53] described a large, 5-center, randomized, double-blind, placebo-controlled study of the efficacy of Ginkgo in reducing the incidence of AD and all-cause dementia in cognitively intact elderly subjects (*n* = 2587) and those with MCI (*n* = 482). The subjects in this study were assessed every 6 months for a median of 6.1 years. Only 6.3% of subjects dropped out or were lost to follow up. Incident dementia was observed to develop in 277 persons receiving Ginkgo and 246 persons receiving placebo; almost all cases were classified as possible or probable AD with or without accompanying cerebrovascular disease. There were no benefits with Ginkgo on any of the outcome measures in either of the subgroups in the study. Ginkgo, therefore, is unlikely to be effective in the primary prevention of AD and other dementias.

## THE TREATMENT OF MILD COGNITIVE IMPAIRMENT

### Donepezil and vitamin E for mild cognitive impairment

Alzheimer's disease does not start overnight; there is a gradual transition from a presumably normal cognitive state through a state of mild cognitive impairment to the clinical syndrome of Alzheimer's disease. Therefore, drugs which improve cognitive performance and slow the progression of Alzheimer's disease should, logically, convey benefits during the preclinical stages of the disease, as well.

Donepezil, a cholinesterase inhibitor, is an established treatment for Alzheimer's disease. Vitamin E, an antioxidant, theoretically retards neurodegeneration and may slow the progression to Alzheimer's disease by targeting very early biological changes. There is also some evidence to suggest that plasma tocopherol levels may be associated with cognitive impairment.[54] Petersen *et al.*[55] therefore conducted a 69-center, North American, randomized, double-blind, placebo-controlled comparison of vitamin E and donepezil in subjects with the mild cognitive impairment.

The sample comprised 769 subjects, aged 55-90 years, who met defined criteria for an insidious onset, gradually progressive, amnestic subtype of mild cognitive impairment. These subjects were randomized to receive vitamin E (2000 IU/day), donepezil (10 mg/day) or placebo for three years. About 30% of the subjects dropped out of the study; the drop out rate was similar across the 3 groups. A total of 212 subjects developed possible or probable NINCDS-ADRDA Alzheimer's disease at the study endpoint; of these, 76% were ApoE epsilon 4 carriers.

Peterson *et al.* found that donepezil significantly retarded the progression to Alzheimer's disease during the first year of treatment but not at the 3-year study endpoint; however, among carriers of one or more ApoE epsilon 4 alleles, the advantage for donepezil was evident throughout the 3-year study. Vitamin E did not retard the progression to Alzheimer's disease at any time point either in the whole sample or in ApoE epsilon 4 alleles. Vitamin E improved cognitive functioning on a few neuropsychological measures, and donepezil improved functioning on many measures; however, the benefits were confined to the first 18 months of the study. Adverse events with donepezil conformed to the known profile of the drug, and included muscle cramps, gastrointestinal symptoms, and sleep disturbances.

The rate of progression to clinically diagnosable Alzheimer's disease is 10%-15% per year among persons with the amnestic form of mild cognitive impairment, relative to a rate of 1%-2% in normal elderly persons.[8] In this study, the overall rate of progression from mild cognitive impairment to Alzheimer's disease was 16% per year. Presence of the ApoE epsilon 4 gene is known to accelerate the transition from mild cognitive impairment to Alzheimer's disease; the results of this study show that donepezil may have preferential prophylactic usefulness in carriers of this gene. A matter of concern is the loss of benefit with donepezil after 12 (progression to Alzheimer's disease) to 18 (neuropsychological measures) months. If these subjects were to later progress to Alzheimer's disease, would benefits with donepezil reappear? Logically, it does not seem likely. Then, should donepezil be stopped and later resumed? But such a strategy could place the subject at risk of accelerated deterioration. There is, therefore, a pressing need for very long-term studies in this field.

A Cochrane review of the use of vitamin E in mild to moderate Alzheimer's disease and MCI highlighted this dearth of strong evidence, though the data was suggestive of some efficacy.[56]

### Cognitive rehabilitation may benefit mild cognitive impairment

In patients with amnestic MCI, a single skill such as calendar organization on a memory notebook system can help compensate for memory loss and improve functional ability. Such a strategy was found to have a modest effect size and also resulted in improvements in mood and self-confidence. However, the sample size in this study was small and there was no placebo control.[57]

There is also preliminary evidence that improvement in one domain may generalize. In a study of training of explicit memory using face-name pairs, subjects were taught to identify a visual stimulus, link a phonological cue to it and recall a name. The significant improvement in recognition accuracy was also found on the untrained stimulus, though to a lesser degree. The small sample size, lack of randomization and absence of a placebo control are limitations of the study.[58]

Multicomponent rehabilitative strategies have also been found effective in MCI. In a study that compared cognitive rehabilitative intervention in patients with MCI and mild AD against a wait-listed MCI group, the multicomponent rehabilitation package (which included activity planning, self-assertiveness training, relaxation techniques, stress management, use of external memory aids, memory training, and motor exercise) was found to improve verbal and non-verbal episodic memory, mood, and functional ability. The practice effect was evident on verbal episodic memory but not on functional ability or mood.[59]

Computer-based cognitive rehabilitation was evaluated in a single-blind trial involving patients with AD and MCI randomized to either computer exercises (12 sessions) or control condition (12 semi-structured interviews). Preliminary analysis revealed that, at 9-month follow up, a significant decline in performance was seen only for the control group as compared with baseline and 3-month follow-up scores.[60] Computer-based retraining was also found to add specific benefits to mood and functional ability in a community rehabilitative program.[61]

## THE PREVENTION OF THE PROGRESSION OF ALZHEIMER'S DISEASE

Many treatments have been described to improve cognition in elderly subjects with and without cognitive deficit states. Certain of these have been suggested to halt or even reverse neurodegeneration or the processes that predispose thereto. Most of these treatments target the production or clearance of amyloid-beta with the assumption that the accumulation of amyloid-beta in the brain predisposes to neurodegeneration and dementia. Such treatments include immunotherapies; secretase inhibitors; selective amyloid beta-42-lowering agents; statins; anti-amyloid beta aggregation agents; peroxisome proliferator-activated receptor-gamma agonists; and others. Drugs that have reached phase III clinical trials include those that selectively target amyloid beta-42 production (e.g. tarenflurbil), enhance the activity of alpha-secretase (e.g., statins), or block amyloid beta aggregation (e.g., transiposate).[62,63] Very recently, a gamma-secretase inhibitor (LY450139) was shown to be well tolerated in a Phase 2 safety trial.[64]

Among the immunological approaches, treatments that prevent the conversion of amyloid-beta into pathological forms and treatments that accelerate clearance of amyloid-beta are presently in development. More than ten such approaches to active and passive immunotherapy are under investigation in clinical trials; the challenge is to establish a favorable balance between the risks associated with the induction of an autoimmune reaction and the benefits associated with the immunological clearance of a potentially harmful endogenous protein.[65]

From a public health perspective, the future of psychopharmacology for Alzheimer's disease lies in prevention, not in treatment. Nevertheless, disease-modifying treatments are important in patients with established disease. This section therefore examines certain treatments with suggested disease-modifying potential. At present, all of these treatments are experimental.

### PBT2 and Alzheimer's disease

In Alzheimer's disease, the conversion of the amyloid-beta peptide from a physiological, water-soluble, monomeric form into neurotoxic oligomeric and fibrillar forms is an undesirable event. This is because the most toxic forms of amyloid-beta are thought to be oligomers, and dimers might be the smallest neurotoxic species.[65]

PBT2 is a metal-protein attenuating compound that reduces the copper- and zinc-mediated toxic oligomerization of amyloid-beta. Animal data from transgenic mouse models of Alzheimer's disease suggest that PBT2 may be beneficial in Alzheimer's patients, and early clinical studies indicate that the compound is safe for human use. With this background, Lannfelt *et al.*[66] conducted an industry-initiated, 12-week, 15-center, randomized, double-blind, placebo-controlled study of PBT2 in community-dwelling patients with Alzheimer's disease.

The Phase IIa clinical trial was conducted in Sweden and Australia. The sample comprised 78 men and women, age > 55 years, who were diagnosed with early Alzheimer's disease. The mean age of the sample was 72 years. The sample was 50% female. ApoE epsilon-4 positivity characterized 76% of the patients. All patients had an MMSE score of 20-26 (mean, 23) or an ADAS-cog score of 10-25 (mean, 19); a modified Hachinski score of 4 points or less; and CT or MRI findings that were consistent with Alzheimer's disease. All patients had also been receiving a stable dose of donepezil, galantamine, or rivastigmine for at least the past 4 months. These patients were randomized to receive PBT2 50 mg/day (*n* = 20), PBT2 250 mg/day (*n* = 29), or placebo (*n* = 29).

CSF and plasma markers of Alzheimer's disease were examined; these included amyloid-beta42, amyloid-beta40, total tau, and P-tau levels. The MMSE, ADAS-cog, and a neuropsychological test battery were also examined as outcome variables. Almost all patients (*n* = 74; 95%) completed the 12-week study.

PBT2 250 mg/day significantly reduced CSF amyloid-beta42 protein; the trend for the dose-dependence of this effect was also significant. PBT2 did not affect other biomarkers of Alzheimer's disease or serum copper and zinc concentrations. Patients who received PBT2 showed improved performance on the category fluency and Trails B tests; however, there were no other differences between PBT2 and placebo in the neuropsychological test battery administered or on MMSE and ADAS-cog. Slightly more than half of the sample (54%) experienced at least one treatment-emergent adverse event; however, there were no differences between groups. PBT2 was not associated with serious adverse events. Although these data are preliminary, they suggest that PBT2 may hold promise as a disease-modifying treatment for Alzheimer's disease.

### Etanercept, TNF-alpha and Alzheimer's disease

Tumor necrosis factor-alpha (TNF-alpha) is an inflammatory and immunomodulatory cytokine. In the brain, TNF-alpha acts as a gliotransmitter which regulates synaptic functioning in neural networks. However, TNF-alpha also increases the expression of interleukin-1, which in turn increases the production of the precursors necessary for the formation of amyloid plaques, neurofibrillary tangles, and Lewy bodies. Thus, chronically elevated TNF-alpha levels can result in neurodegeneration.[67]

Etanercept is a USA Food and Drug Administration (FDA)-approved TNF-alpha inhibitor used for the treatment of rheumatoid arthritis and other systemic diseases associated with inflammation. A recent report suggested that etanercept may convey dramatic benefits in patients with Alzheimer's disease.[68]

The patient was an 81-year-old doctor with late-onset Alzheimer's disease. He was treated with weekly injections of perispinal etanercept. Physicians, family members, friends, and objective tests all attested to considerable improvements in memory after the etanercept injections. Certain of these improvements began within minutes of the injections. For example, After the injection, the patient was unable to state the date, day of the week, year, place, city, or state. After injection, he was able to name the day, month, and state. The improvements lasted for at least seven weeks with the weekly injections.

Etanercept needs to be administered by perispinal injection because it is a large molecule which does not cross the blood-brain barrier. Griffin[67] referred to an unpublished, nonblind, 6-month pilot study of etanercept in 15 patients with probable Alzheimer's disease. These patients were treated weekly with a perispinal injection of etanercept. There was rapid-onset, sustained improvement observed in cognitive functions. These findings support the report of Tobinick and Gross.[68] Whereas etanercept does not cure Alzheimer's disease, it may halt the progression of the disease. Furthermore, the dramatically rapid onset of benefits suggests that acute synaptic mechanisms may mediate many of the cognitive impairments of Alzheimer's disease, and that these mechanisms can be antagonized with immediate therapeutic results.

### Tarenflurbil for Alzheimer's disease

Tarenflurbil selectively targets amyloid-beta42 production. Wilcock *et al.*[69] described an industry-sponsored, 12-month, multicenter, phase II, randomized, double-blind, placebo-controlled trial of tarenflurbil (R-flurbiprofen) in patients with Alzheimer's disease. The sample comprised 210 community-dwelling patients with mild to moderate Alzheimer's disease. Baseline MMSE scores were in the 15-26 point range. These patients were randomized to receive tarenflurbil 800 mg/day (n = 69), tarenflurbil 1600 mg/day (n = 70) or placebo (n = 71) for 12 months. In a 12-month extension of the primary study, patients who had received tarenflurbil continued to receive the same dose, and patients who had received placebo were randomized to tarenflurbil at 800 mg/day or 1600 mg/day.

In patients with mild Alzheimer's disease (MMSE, 20-26), at 12 months, the 1600 mg/day dose of tarenflurbil was associated with less decline in activities of daily living (effect size, 0.45) and global functioning (effect size, 0.42) than placebo. However, cognitive decline did not differ significantly between tarenflurbil 1600 mg/day and placebo groups. In patients with moderate Alzheimer's disease (MMSE, 15-19), at 12 months, the 1600 mg/day dose did not influence activities of daily living or cognition but significantly worsened global functioning (effect size, 1.08). Patients with mild Alzheimer's disease who received tarenflurbil 1600 mg/day for 24 months had less cognitive decline, better activities of daily living, and better global functioning than those who received placebo for the first 12 months and either placebo or tarenflurbil 1600 mg/day thereafter. Tarenflurbil was generally well-tolerated and was associated with a placebo level of adverse effects.

These preliminary data suggest that tarenflurbil 1600 mg/day is well tolerated and across a 2-year period, may benefit cognition, activities of daily living, and global functioning in patients with mild Alzheimer's disease. Whether tarenflurbil has no effect on or worsens moderately severe Alzheimer's disease is uncertain. The pharmacokinetics of tarenflurbil were described by Galasko *et al.*[70]

Disappointingly, on June 30, 2008, Myriad Genetics, the developers of tarenflurbil, announced that an 18-month, phase III trial in patients with mild Alzheimer's disease failed to demonstrate a statistically significant advantage for tarenflurbil on either of the two primary endpoints: cognition and activities of daily living. The company added that it would discontinue the development of the drug. The announcement is available here: http://www.myriad.com/news/release/1170283

### The Alzheimer's vaccine

Approaches towards the development of a vaccine against Alzheimer's disease have been based on two strategies: active immunotherapy using the pre-aggregated synthetic amyloid-beta42 preparation AN1792 vaccine (QS-21) and passive immunization using injections of already prepared polyclonal anti-beta amyloid antibodies (intravenous immunoglobulin).

The candidate Alzheimer's vaccine AN1792 was found to reduce amyloid-beta42 plaque burden and preserve cognitive function in amyloid precursor protein transgenic mice. The phase IIa trial in humans was discontinued because of the occurrence of meningoencephalitis in 6% of the patients. However, the analysis of data revealed that the performance of patients who received the vaccine improved significantly on the neuropsychological test battery.[71] In a 6-year follow up of 9 Phase I patients, postmortem analysis showed that the vaccine was associated with greater clearing of beta amyloid plaques; however, the survival or time to progression to severe dementia was not significantly improved in these patients.[72]

### Growth hormone secretagogue

There is a physiological decline of the growth hormone (GH)/insulin-like growth factor-I (IGF-I) axis with ageing. IGF-I plays a crucial role in neurodevelopment and facilitates survival of neurons. The GH/IGF-I axis has also been implicated in pathophysiology of cognitive deficits during aging. In this regard, the growth hormone secretagogue MK-677 (ibutamoren mesylate), is a potent inducer of IGF-1 secretion and has hence been suggested to slow the rate of progression of AD. However, a 12-month, randomized, double-blind, multicenter study which compared MK-677 with placebo in patients (*n* = 563) with mild to moderate AD found no significant difference between groups on measures of cognition and activities of daily living (ADL).[73] Perhaps treatments such as this need to be tested earlier during the course of the disease; once the Alzheimer pathology is established, induction of IGF-1 may serve little purpose.

## THE TREATMENT OF COGNITIVE SYMPTOMS IN ELDERLY PERSONS WITH AND WITHOUT COGNITIVE SYNDROMES

The cholinergic hypothesis of dementia[74] implicates degeneration of cholinergic neurons in the basal forebrain as a central pathological feature of Alzheimer's disease. Drug development for dementia has hence focused on correcting the cholinergic deficit by utilizing one of three strategies: the supply of precursor agents in order to increase the production of acetylcholine (ACh); cholinergic agonism to compensate for ACh deficit and cholinesterase inhibition to increase the availability of ACh.

Cholinergic drugs which currently have USA FDA approval for Alzheimer's disease are tacrine, donepezil, rivastigmine, and galantamine. Memantine, which targets NMDA receptors, is another FDA-approved drug. Experimental treatments for cognitive deficits include ginkgo biloba extract, phototherapy, melatonin, vitamin E, and Transcutaneous Electrical Nerve Stimulation (TENS).

Among earlier treatments used for Alzheimer's disease, hydergine (ergoloid mesylates) was held in esteem; however, recommendations for hydergine are difficult to make because the clinical trials had many methodological limitations, including the absence of application of formal diagnostic criteria for dementia.[75] Piracetam, a putative nootropic agent, has been studied in randomized, double-blind, placebo-control trials, but the results in dementia have been mixed.[76] In the section that follows, we examine results with a number of experimental treatments.

### Dimebon may improve cognition in Alzheimer's disease

Dimebon (dimebolin) is a 25-year-old Russian antihistaminic drug. Dimebon dampens NMDA receptor currents in some neurons, and increases AMPA receptor activity.[77] Furthermore, neurotoxins such as amyloid-beta open mitochondrial permeability transition pores which regulate transport of ions and peptides in and out of mitochondria, and which are associated with a general mechanism for maintaining calcium ion homeostasis and apoptosis; dimebon blocks this action of amyloid-beta.[78]

In a double-blind, randomized controlled study conducted at 11 Russian sites, Doody *et al.*[79] recruited 183 patients with mild-to-moderate Alzheimer's disease; MMSE scores were in the 10-24 point range. These patients were randomized to receive oral dimebon (60 mg/day; *n* = 89), or placebo (*n* = 94) for 26 weeks. Eleven patients dropped out from the dimebon group and 17 from the placebo group. At the 26-week study endpoint, dimebon was associated with a small but statistically significant improvement in ADAS-cog scores, while placebo was associated with deterioration. Dimbon-treated patients showed better cognitive performances than placebo-treated patients. Dimebon was well tolerated and was generally associated with a placebo level of adverse effects; however, dry mouth (14%) and depressed mood (14%) were common adverse events.

### Herbal medicines may attenuate cognitive impairment

Many studies have investigated the efficacy of herbal medicines in cognitive deficit disorders.

In a systematic review and meta-analysis, Birks and Evans (50) observed that many studies have found cognitive benefits with a standardized extract of Ginkgo biloba after intermediate- to long-term administration to patients with Alzheimer's disease; however, as much of the literature is of poor methodological quality, these authors concluded that the clinical value of Ginkgo is unconvincing.

A large number of Indian herbal preparations and formulations have been suggested to have procognitive properties in animal models; however, the applicability of such research to clinical contexts is suspect.[80]

The Chinese herbal preparation Naohuandan was shown to be comparable to piracetam in improving cognitive function in a randomized controlled trial of patients with dementia.[81] Shenyin Oral Liquid (SOL)[82] and Modified Wuzi Yanzong Granule[83] were shown to improve MCI in placebo-controlled trials; improvement on the memory quotient correlated with superoxide dismutase (SOD) activity, suggesting a possible mechanism of action.[83]

In an Indian study, the herbal formulation Memorin (Phyto-Pharma, Kolhapur) was evaluated in a 3-month, randomized, double-blind, placebo-controlled study in 45 subjects with DSM-IV age-related cognitive decline. All subjects completed a battery of neuropsychological tests that assessed visual and verbal memory, visuospatial skills, and perceptuomotor functioning. In the Memorin group, there was significant improvement on most tests; however, improvement in many of the memory tasks was confined to males. Age did not significantly influence the results. In contrast, in placebo-treated subjects there was little therapeutic gain.[84]

Such positive results notwithstanding, it should be kept in mind that sample sizes were small and study methodologies were mostly poor in quality. No conclusions or recommendations for practice can be made from the current body of research.

### Omega-3 fatty acids do not benefit cognition in the elderly

Epidemiological studies suggest that fish intake reduces the risk of age-related cognitive decline and Alzheimer's disease. For example, Morris *et al.*[17] found that the dietary intake of omega-3 fatty acids and an at least once-weekly consumption of fish decreased the risk of Alzheimer's disease. Huang *et al.*[18] found that elderly subjects who ate fatty fish more often than twice a week had a 41% lower risk of Alzheimer's disease; though, the benefits were limited to subjects without the ApoE epsilon 4 allele. Nurk *et al.*[21] showed that elderly subjects who ate more fish and fish products had better cognitive performance across a range of cognitive domains; the effect was dose-dependent. Van Gelder *et al.*[22] showed that elderly subjects who ate fish and who consumed more omega-3 fatty acids in their diet suffered less cognitive decline across 5 years; the effect was again dose-dependent.

The benefits of fish are believed to arise from the omega-3 polyunsaturated fatty acid content. Van de Rest *et al.*[85] therefore conducted a 26-week, randomized, double-blind, placebo-controlled trial to identify the cognitive benefits, if any, of the fish oils EPA and DHA in elderly subjects. The sample comprised 302 community-dwelling subjects aged 65 years and older. Subjects were cognitively intact at baseline, and all had an MMSE score of 22 and above. The mean age of the sample was about 70 years. The sample was 55% male. These subjects were randomized to receive EPA-DHA 1800 mg/day, EPA-DHA 400 mg/day, or placebo for 26 weeks. Cognitive performance was assessed using an extensive neuropsychological test battery; the cognitive domains that were tested included attention, sensorimotor speed, memory, and executive function. Plasma concentrations of EPA-DHA increased by 238% in the high-dose group and by 51% in the low-dose group. Overall, neither low nor high-dose groups showed cognitive changes that differed from the placebo group in any of the tested cognitive domains. As a further discouragement, chronic (12-week) dosing with EPA (2 g/day) has been shown to increase weight as well as prolong the bleeding time;[86] these risks should be factored into therapeutic plans.

Epidemiological studies report on behavior across years or even decades. Therefore, if fish oil supplementation is at all beneficial, it may need to be sustained for long periods for cognitive benefits to be detectable and may need to be implemented as a preventive measure rather than as a treatment; that is, when the biological capacity to respond to beneficial influences is greater, rather than at a later age when biological systems are losing their flexibility or have developed irreversible pathological changes.

### Ventriculoperitoneal shunt is unhelpful for Alzheimer's disease

The neuropathological hallmarks of Alzheimer's disease include amyloid plaques and neurofibrillary tangles; these are formed from amyloid peptide and tau protein, respectively. If amyloid and tau are more efficiently cleared from the CNS, there is a theoretically lower likelihood of their deposit in the brain, and hence a lower risk of resultant neurodegeneration. Encouraged by the positive results of a pilot study,[87] Silverberg *et al.*[88] tested an intriguing hypothesis in a 9-month, randomized, double-blind, controlled trial: that draining CSF through a ventriculoperitoneal shunt would reduce the macromolecular load on the CNS and benefit patients with Alzheimer's disease.

The sample comprised 215 subjects with probable Alzheimer's disease diagnosed using the NINDS-ADRDA criteria. Illness in these patients ranged from mild to severe. All patients underwent surgery for the implant of a low-flow ventriculoperitoneal shunt which was open (active group) or occluded (control group). CSF was longitudinally sampled in both groups.

The surgical procedure and the shunt were associated with 12 CNS infections. Some of these infections were temporally associated with the CSF sampling. All infections were treated successfully.

A planned interim analysis found that the active and control groups did not differ on measures of dementia, deterioration, and levels of amyloid and tau precursors in CSF. The study was prematurely halted because it appeared to be an exercise in futility.

### Anticholinergic drugs impair anticholinesterase efficacy in dementia

Patients with dementia frequently suffer from comorbid conditions; some of these conditions may require treatment with medications that have anticholinergic action. What effects do anticholinergic medications have in dementia? The issue was investigated by Sink *et al.*[89]

The sample was drawn from 3536 nursing home residents aged 65 years and older. Of these, 376 were receiving a cholinesterase inhibitor for dementia and an anticholinergic drug (either oxybutynin or tolterodine) for urinary frequency or incontinence. Patients who were taking other anticholinergic drugs were excluded from the sample.

Assessments were made using the Minimum Data Set (MDS). Cognitive functioning was assessed using the MDS Cognition Scale (scored 0-10) and activities of daily living were assessed using seven pertinent items in the MDS (scored 0-28). Potential covariates included age, sex, race, number of medications, and the Charlson Comorbidity Index score.

In patients who were in the top quartile of functioning in their activities of daily living, the use of anticholinergic medication was associated with a 50% greater rate in decline per 3-month period across a time span of two years. The activities of daily living score decreased by an average of 1.08 points per quarter in patients receiving a cholinesterase inhibitor alone, as compared with 1.62 points in those receiving a cholinesterase inhibitor along with oxybutynin or tolterodine. There was no excess decline in cognition or in activities of daily living associated with dual therapy in patients with lower levels of functioning. Thus, in elderly subjects with dementia who are functioning at higher levels in everyday life, the use of anticholinergic drugs accelerates deterioration in activities of daily living.

Although this study examined only the interaction between cholinesterase inhibitors and oxybutynin or tolterodine, it is likely that the findings will be similar whatever the medication used for dementia, and whatever the anticholinergic drug used. This is because anticholinergic drugs are well known to impair cognition. The magnitude of impairment will likely depend on the dose and potency of the anticholinergic treatment.

In this study, why did the anticholinergic drugs impair only activities of daily living and only in patients functioning at higher levels? One explanation is that the assessment tool was not sensitive enough to identify impairments in cognition. Another explanation is that patients at lower levels of functioning were too impaired to be further impaired by the anticholinergic drugs.

Would the excess deterioration halt or reverse with withdrawal of the anticholinergic medication? This is a question worth addressing in future studies. Whereas it seems logical that a drug effect will last only for as long as the drug is used, it should be recognized that dementia patients who lose abilities may take time to recover the abilities, if at all the recovery occurs.

## CONCLUDING NOTES

There has been an explosion of research into intuitive and innovative experimental treatments that seek to prevent cognitive impairment, improve cognition, or prevent deterioration in normal elderly subjects, and those with mild cognitive impairment or Alzheimer's disease. As we have observed in our article, many of the approaches have led to a dead end. However, other approaches appear promising. It is likely that, during the next five years, dramatic developments in the described fields may change the way clinicians approach the problem of protecting cognition in the elderly, and preventing cognitive decline in already impaired persons.

## References

[1] Kokmen E, Beard CM, O'Brien PC, Offord KP, Kurland LT (1993). Is the incidence of dementing illness changing? A 25-year time trend study in Rochester, Minnesota (1960-1984). Neurology.

[2] Drachman DA (1994). If we live long enough, will we all be demented?. Neurology.

[3] Plassman BL, Langa KM, Fisher GG, Heeringa SG, Weir DR, Ofstedal MB (2007). Prevalence of dementia in the United States: the aging, demographics, and memory study. Neuroepidemiology.

[4] Ferri CP, Prince M, Brayne C, Brodaty H, Fratiglioni L, Ganguli M (2005). Global prevalence of dementia: a Delphi consensus study. Lancet.

[5] Vas CJ, Pinto C, Panikker D, Noronha S, Deshpande N, Kulkarni L (2001). Prevalence of dementia in an urban Indian population. Int Psychogeriatr.

[6] Shaji S, Bose S, Verghese A (2005). Prevalence of dementia in an urban population in Kerala, India. Br J Psychiatry.

[7] Rajkumar S, Kumar S, Thara R (1997). Prevalence of dementia in a rural setting - a report from India. Int J Geriatr Psychiatry.

[8] Petersen RC, Smith GE, Waring SC, Ivnik RJ, Tangalos EG, Kokmen E (1999). Mild cognitive impairment: clinical characterization and outcome. Arch Neurol.

[9] Morris JC (2006). Mild cognitive impairment is early-stage Alzheimer disease: time to revise diagnostic criteria. Arch Neurol.

[10] Petersen RC, O'Brien J (2006). Mild cognitive impairment should be considered for DSM-V. J Geriatr Psychiatry Neurol.

[11] Ikeda M (2004). Nippon Ronen Igakkai Zasshi. Epidemiology of Mild Cognitive Impairment (MCI) among the community-dwelling elderly-findings from the Nakayama study. Nippon Ronen Igakkai Zasshi.

[12] Ganguli M, Dodge HH, Shen C, DeKosky ST (2004). Mild cognitive impairment, amnestic type: an epidemiologic study. Neurology.

[13] Palmer K, Bäckman L, Winblad B, Fratiglioni L (2008). Mild cognitive impairment in the general population: occurrence and progression to Alzheimer disease. Am J Geriatr Psychiatry.

[14] Das SK, Bose P, Biswas A, Dutt A, Banerjee TK, Hazra AM (2007). An epidemiologic study of mild cognitive impairment in Kolkata, India. Neurology.

[15] Birks J (2006). Cholinesterase inhibitors for Alzheimer's disease. Cochrane Database Syst Rev.

[16] Morris MC, Evans DA, Tangney CC, Bienias JL, Wilson RS (2005). Fish consumption and cognitive decline with age in a large community study. Arch Neurol.

[17] Morris MC, Evans DA, Bienias JL, Tangney CC, Bennett DA, Wilson RS (2003). Consumption of fish and omega-3 fatty acids and risk of incident Alzheimer disease. Arch Neurol.

[18] Huang TL, Zandi PP, Tucker KL, Fitzpatrick AL, Kuller LH, Fried LP (2005). Benefits of fatty fish on dementia risk are stronger for those without ApoE epsilon4. Neurology.

[19] Dai Q, Borenstein AR, Wu Y, Jackson JC, Larson EB (2006). Fruit and vegetable juices and Alzheimer's disease: the Kame Project. Am J Med.

[20] Barberger-Gateau P, Raffaitin C, Letenneur L, Berr C, Tzourio C, Dartigues JF (2007). Dietary patterns and risk of dementia: the Three-City cohort study. Neurology.

[21] Nurk E, Drevon CA, Refsum H, Solvoll K, Vollset SE, Nygerd O (2007). Cognitive performance among the elderly and dietary fish intake: the Hordaland Health Study. Am J Clin Nutr.

[22] van Gelder BM, Tijhuis M, Kalmijn S, Kromhout D (2007). Fish consumption, n-3 fatty acids, and subsequent 5-y cognitive decline in elderly men: the Zutphen Elderly Study. Am J Clin Nutr.

[23] Dullemeijer C, Durga J, Brouwer IA, van de Rest O, Kok FJ, Brummer RJ (2007). Omega-3 fatty acid proportions in plasma and cognitive performance in older adults. Am J Clin Nutr.

[24] Beydoun MA, Kaufman JS, Satia JA, Rosamond W, Folsom AR (2007). Plasma n-3 fatty acids and the risk of cognitive decline in older adults: the Atherosclerosis Risk in Communities Study. Am J Clin Nutr.

[25] Ng TP, Feng L, Niti M, Kua EH, Yap KB (2008). Tea consumption and cognitive impairment and decline in older Chinese adults. Am J Clin Nutr.

[26] Stern Y (2006). Cognitive reserve and Alzheimer disease. Alzheimer Dis Assoc Disord.

[27] Ravaglia G, Forti P, Lucicesare A, Pisacane N, Rietti E, Bianchin M (2008). Physical activity and dementia risk in the elderly: findings a prospective Italian study. Neurology.

[28] Larson EB, Wang L, Bowen JD, McCormick WC, Teri L, Crane P (2006). Exercise is associated with reduced risk for incident dementia among persons 65 years of age and older. Ann Intern Med.

[29] Stranahan AM, Khalil D, Gould E (2007). Running induces widespread structural alterations in the hippocampus and entorhinal cortex. Hippocampus.

[30] Lange-Asschenfeldt C, Kojda G (2008). Alzheimer's disease, cerebrovascular dysfunction and the benefits of exercise: From vessels to neurons. Exp Gerontol.

[31] van Uffelen JG, Chinapaw MJ, van Mechelen W, Hopman-Rock M (2008). Walking or vitamin B for cognition in older adults with mild cognitive impairment? A randomised controlled trial. Br J Sports Med.

[32] Forbes D, Forbes S, Morgan DG, Markle-Reid M, Wood J, Culum I (2008). Physical activity programs for persons with dementia. Cochrane Database Syst Rev.

[33] Friedland RP, Fritsch T, Smyth KA, Koss E, Lerner AJ, Chen CH (2001). Patients with Alzheimer's disease have reduced activities in midlife compared with healthy control-group members. Proc Natl Acad Sci U S A.

[34] Wilson RS, Mendes De Leon CF, Barnes LL, Schneider JA, Bienias JL, Evans DA (2002). Participation in cognitively stimulating activities and risk of incident Alzheimer disease. JAMA.

[35] Ball K, Berch DB, Helmers KF, Jobe JB, Leveck MD, Marsiske M (2002). Effects of cognitive training interventions with older adults: a randomized controlled trial. JAMA.

[36] Willis SL, Tennstedt SL, Marsiske M, Ball K, Elias J, Koepke KM (2006). Long-term effects of cognitive training on everyday functional outcomes in older adults. JAMA.

[37] Wolozin B, Wang SW, Li NC, Lee A, Lee TA, Kazis LE (2007). Simvastatin is associated with a reduced incidence of dementia and Parkinson's disease. BMC Med.

[38] Sparks DL, Kryscio RJ, Sabbagh MN, Connor DJ, Sparks LM, Liebsack C (2008). Reduced risk of incident AD with elective statin use in a clinical trial cohort. Curr Alzheimer Res.

[39] Cramer C, Haan MN, Galea S, Langa KM, Kalbfleisch JD (2008). Use of statins and incidence of dementia and cognitive impairment without dementia in a cohort study. Neurology.

[40] Lichtenthaler SF, Haass C (2004). Amyloid at the cutting edge: activation of alpha-secretase prevents amyloidogenesis in an Alzheimer disease mouse model. J Clin Invest.

[41] Rea TD, Breitner JC, Psaty BM, Fitzpatrick AL, Lopez OL, Newman AB (2005). Statin use and the risk of incident dementia: the Cardiovascular Health Study. Arch Neurol.

[42] Zandi PP, Sparks DL, Khachaturian AS, Tschanz J, Norton M, Steinberg M (2005). Do statins reduce risk of incident dementia and Alzheimer disease? The Cache County Study. Arch Gen Psychiatry.

[43] Kessing LV, Nilsson FM (2003). Increased risk of developing dementia in patients with major affective disorders compared to patients with other medical illnesses. J Affect Disord.

[44] Muñoz-Montaño JR, Lim F, Moreno FJ, Avila J, Díaz-Nido J (1999). Glycogen Synthase Kinase-3 Modulates Neurite Outgrowth in Cultured Neurons: Possible Implications for Neurite Pathology in Alzheimer's Disease. J Alzheimers Dis.

[45] Lovestone S, Davis DR, Webster MT, Kaech S, Brion JP, Matus A (1999). Lithium reduces tau phosphorylation: effects in living cells and in neurons at therapeutic concentrations. Biol Psychiatry.

[46] Phiel CJ, Wilson CA, Lee VM, Klein PS (2003). GSK-3alpha regulates production of Alzheimer's disease amyloid-beta peptides. Nature.

[47] Nunes PV, Forlenza OV, Gattaz WF (2007). Lithium and risk for Alzheimer's disease in elderly patients with bipolar disorder. Br J Psychiatry.

[48] Kessing LV, Søndergård L, Forman JL, Andersen PK (2008). Lithium treatment and risk of dementia. Arch Gen Psychiatry.

[49] Dunn N, Holmes C, Mullee M (2005). Does lithium therapy protect against the onset of dementia?. Alzheimer Dis Assoc Disord.

[50] Birks J, Grimley Evans J (2007). Ginkgo biloba for cognitive impairment and dementia. Cochrane Database Syst Rev.

[51] Dodge HH, Zitzelberger T, Oken BS, Howieson D, Kaye J (2008). A randomized placebo-controlled trial of Ginkgo biloba for the prevention of cognitive decline. Neurology.

[52] McCarney R, Fisher P, Iliffe S, van Haselen R, Griffin M, van der Meulen (2008). Ginkgo biloba for mild to moderate dementia in a community setting: a pragmatic, randomised, parallel-group, double-blind, placebo-controlled trial. Int J Geriatr Psychiatry.

[53] DeKosky ST, Williamson JD, Fitzpatrick AL, Kronmal RA, Ives DG, Saxton JA (2008). Ginkgo biloba for prevention of dementia: a randomized controlled trial. JAMA.

[54] Ravaglia G, Forti P, Lucicesare A, Pisacane N, Rietti E, Mangialasche F (2008). Plasma tocopherols and risk of cognitive impairment in an elderly Italian cohort. Am J Clin Nutr.

[55] Petersen RC, Thomas RG, Grundman M, Bennett D, Doody R, Ferris S (2005). Vitamin E and donepezil for the treatment of mild cognitive impairment. N Engl J Med.

[56] Isaac MG, Quinn R, Tabet N (2008). Vitamin E for Alzheimer's disease and mild cognitive impairment. Cochrane Database Syst Rev.

[57] Greenaway MC, Hanna SM, Lepore SW, Smith GE (2008). A behavioral rehabilitation intervention for amnestic mild cognitive impairment. Am J Alzheimers Dis Other Demen.

[58] Hampstead BM, Sathian K, Moore AB, Nalisnick C, Stringer AY (2008). Explicit memory training leads to improved memory for face-name pairs in patients with mild cognitive impairment: results of a pilot investigation. J Int Neuropsychol Soc.

[59] Kurz A, Pohl C, Ramsenthaler M, Sorg C (2008). Cognitive rehabilitation in patients with mild cognitive impairment. Int J Geriatr Psychiatry.

[60] Galante E, Venturini G, Fiaccadori C (2007). Computer-based cognitive intervention for dementia: preliminary results of a randomized clinical trial. G Ital Med Lav Ergon.

[61] Talassi E, Guerreschi M, Feriani M, Fedi V, Bianchetti A, Trabucchi M (2007). Effectiveness of a cognitive rehabilitation program in mild dementia (MD) and mild cognitive impairment (MCI): a case control study. Arch Gerontol Geriatr.

[62] Christensen DD (2007). Changing the course of Alzheimer's disease: anti-amyloid disease-modifying treatments on the horizon. Prim Care Companion J Clin Psychiatry.

[63] Christensen DD (2007). Alzheimer's disease: progress in the development of anti-amyloid disease-modifying therapies. CNS Spectr.

[64] Fleisher AS, Raman R, Siemers ER, Becerra L, Clark CM, Dean RA (2008). Phase 2 safety trial targeting amyloid beta production with a gamma-secretase inhibitor in Alzheimer disease. Arch Neurol.

[65] Wisniewski T, Konietzko U (2008). Amyloid-beta immunisation for Alzheimer's disease. Lancet Neurol.

[66] Lannfelt L, Blennow K, Zetterberg H, Batsman S, Ames D, Harrison J (2008). Safety, efficacy, and biomarker findings of PBT2 in targeting A-beta as a modifying therapy for Alzheimer's disease: a phase IIa, double-blind, randomised, placebo-controlled trial. Lancet Neurol.

[67] Griffin WS (2008). Perispinal etanercept: Potential as an Alzheimer therapeutic. J Neuroinflammation.

[68] Tobinick EL, Gross H (2008). Rapid cognitive improvement in Alzheimer's disease following perispinal etanercept administration. J Neuroinflammation.

[69] Wilcock GK, Black SE, Hendrix SB, Zavitz KH, Swabb EA, Laughlin MA (2008). Efficacy and safety of tarenflurbil in mild to moderate Alzheimer's disease: a randomised phase II trial. Lancet Neurol.

[70] Galasko DR, Graff-Radford N, May S, Hendrix S, Cottrell BA, Sagi SA (2007). Safety, tolerability, pharmacokinetics, and Abeta levels after short-term administration of R-flurbiprofen in healthy elderly individuals. Alzheimer Dis Assoc Disord.

[71] Gilman S, Koller M, Black RS, Jenkins L, Griffith SG, Fox NC (2005). Clinical effects of Abeta immunization (AN1792) in patients with AD in an interrupted trial. Neurology.

[72] Holmes C, Boche D, Wilkinson D, Yadegarfar G, Hopkins V, Bayer A (2008). Long-term effects of Abeta42 immunisation in Alzheimer's disease: follow-up of a randomised, placebo-controlled phase I trial. Lancet.

[73] Sevigny JJ, Ryan JM, van Dyck CH, Peng Y, Lines CR, Nessly ML (2008). Growth hormone secretagogue MK-677: no clinical effect on AD progression in a randomized trial. Neurology.

[74] Bartus RT, Dean RL, Beer B, Lippa AS (1982). The cholinergic hypothesis of geriatric memory dysfunction. Science.

[75] Olin J, Schneider L, Novit A, Luczak S (2001). Hydergine for dementia. Cochrane Database Syst Rev.

[76] Vernon MW, Sorkin EM (1991). Piracetam. An overview of its pharmacological properties and a review of its therapeutic use in senile cognitive disorders. Drugs Aging.

[77] Grigor'ev VV, Dranyi OA, Bachurin SO (2003). Comparative Study of Action Mechanisms of Dimebon and Memantine on AMPA- and NMDA-Subtypes Glutamate Receptors in Rat Cerebral Neurons. Bull Exp Biol Med.

[78] Bachurin SO, Shevtsova EP, Kireeva EG, Oxenkrug GF, Sablin SO (2003). Mitochondria as a target for neurotoxins and neuroprotective agents. Ann N Y Acad Sci.

[79] Doody RS, Gavrilova SI, Sano M, Thomas RG, Aisen PS, Bachurin SO (2008). Effect of dimebon on cognition, activities of daily living, behaviour, and global function in patients with mild-to-moderate Alzheimer's disease: a randomised, double-blind, placebo-controlled study. Lancet.

[80] Andrade C, Sudha S, Venkataraman BV (2000). Herbal treatments for ECS-induced memory deficits: a review of research and a discussion on animal models. J ECT.

[81] Meng RS, Li QM, Wei CX, Chen B, Liao HY, Zhou YT (2005). Clinical observation and mechanism study on treatment of senile dementia with Naohuandan. Chin J Integr Med.

[82] Zhou RQ, Lin SM, Yuan Q (2007). Clinical study on effect of shenyin oral liquid in treating mild cognitive impairment. Zhongguo Zhong Xi Yi Jie He Za Zhi.

[83] Wang XM, Fu H, Liu GX, Zhu W, Li L, Yang JX (2007). Effect of modified wuzi yanzong granule on patients with mild cognitive impairment from oxidative damage aspect. Chin J Integr Med.

[84] Andrade C, Gowda S, Chaturvedi SK (1998). Treatment of age-related cognitive decline with a herbal formulation: A double-blind study. Indian J Psychiatry.

[85] van de Rest O, Geleijnse JM, Kok FJ, van Staveren WA, Dullemeijer C, OldeRikkert MG (2008). Effect of fish oil on cognitive performance in older subjects: a randomized, controlled trial. Neurology.

[86] Emsley R, Niehaus DJ, Oosthuizen PP, Koen L, Ascott-Evans B, Chiliza B (2008). Safety of the omega-3 fatty acid, eicosapentaenoic acid (EPA) in psychiatric patients: Results from a randomized, placebo-controlled trial. Psychiatry Res.

[87] Silverberg GD, Levinthal E, Sullivan EV, Bloch DA, Chang SD, Leverenz J (2002). Assessment of low-flow CSF drainage as a treatment for AD: results of a randomized pilot study. Neurology.

[88] Silverberg GD, Mayo M, Saul T, Fellmann J, Carvalho J, McGuire D (2008). Continuous CSF drainage in AD: Results of a double-blind, randomized, placebo-controlled study. Neurology.

[89] Sink KM, Thomas J, Xu H, Craig B, Kritchevsky S, Sands LP (2008). Dual use of bladder anticholinergics and cholinesterase inhibitors: long-term functional and cognitive outcomes. J Am Geriatr Soc.

